# Pregnancy outcomes of Q fever: prospective follow-up study on Reunion island

**DOI:** 10.1186/s12879-019-4619-6

**Published:** 2019-11-27

**Authors:** Yoan Mboussou, Julien Jaubert, Sophie Larrieu, Laura Atiana, Florence Naze, Christine Folio, Hanitra Randrianaivo, Antoine Bertolotti, Sandrine Picot, Pierre-Yves Robillard, Malik Boukerrou, Patrick Gérardin

**Affiliations:** 1 Laboratoire de Bactériologie, Virologie et Parasitologie, Centre Hospitalier Universitaire (CHU) de la Réunion, St Pierre, Reunion France; 2 CIRE Ocean Indien, Santé Publique France, French National Public Health Agency, St Denis, Reunion France; 3 Maternité, Pôle Femme Mère Enfant, CHU de la Réunion, St Pierre, Reunion France; 4 Service des Maladies Infectieuses, CHU de la Réunion, St Pierre, Reunion France; 5 INSERM CIC 1410 Epidémiologie Clinique, CHU Réunion, Centre Hospitalier Universitaire, Groupe Hospitalier Sud Réunion, BP 350, 97448 Saint Pierre, Cedex – Reunion France; 6 CEPOI-EA7388, Pôle Femme Mère Enfant, CHU de la Réunion, St Pierre, Reunion France; 7 UM 134 PIMIT Processus Infectieux en Milieu Insulaire Tropical, INSERM 1187, CNRS 9192, IRD 249, Université de La Réunion, CYROI, Ste Clotilde, Reunion France

**Keywords:** Serology, Immunofluorescence, Cohort studies, *Q fever*, *Coxiella burnetii*, Zoonosis, Incidence, Pregnancy, Miscarriage, Stillbirth

## Abstract

**Background:**

Q fever has been associated with perinatal complications. We conducted a prospective follow-up study to assess both the incidence of adverse pregnancy outcomes (APOs) associated with *Coxiella burnetii* infection and the contribution of Q fever to APOs.

**Methods:**

Between May 1 and October 31, 2013, within the regional perinatal health care centre of Saint Pierre, Reunion island, we investigated unexplained miscarriages, stillbirths, preterm births or small-for-gestational age children. Seropositivity for *C. burnetii* antibodies was defined using indirect immunofluorescence for a phase 2 IgG titre ≥1:64. Acute Q fever was defined for a high phase 2 IgG titre ≥1:256 (compatible with recent or active infection) or the detection of *C. burnetii* genome in miscarriage products and placentas. Incidence rate ratios (IRR) for Q fever related APOs (taken as a composite outcome or individually) were assessed using Poisson regression models for dichotomous outcomes controlling major confounders.

**Results:**

Over a 6-month period, 179 pregnant women suspected or diagnosed with an APO were investigated for Q fever, of whom 118 met the definition for an APO. Of these, 19 were seropositive and 10 presented a profile indicative of an acute infection. For three women with an acute Q fever, the chronology between the onset of infection, the APO (2 miscarriages, 1 preterm birth) and the seroconversion suggested causality in the pathogenesis. The cumulative incidence of Q fever related APOs was estimated between 2.2‰ and 5.2‰, whether causality was required or not. Both *C. burnetii* exposure and acute Q fever were independently associated with APOs (IRR 1.55, 95% CI 1.31–1.84; IRR 1.47, 95% CI 1.15–1.89, respectively).

**Conclusions:**

In the endemic context of Reunion island, acute Q fever may lead to APOs. To limit the burden of Q fever on reproduction, pregnant women should be kept away from farms and avoid direct contact with ruminants.

## Background

Q fever is a zoonosis of global public health importance that is caused by *Coxiella burnetii*, an obligate Gram negative intracellular bacterium maintained in wildlife through mammals, birds and arthropods (*e.g,* ticks), serving as reservoirs [1]. Cattle, goats and sheep are the primary sources of human contamination [1, 2]. These animals suffer various reproductive disorders, of which spontaneous abortion (miscarriage), preterm delivery, intrauterine growth restriction and foetal loss may represent an economic burden [1–3]. Human infection is usually acquired through the inhalation of contaminated aerosols from infected animals that contaminate the environment through excretion of bacteria in large amounts in by-products of childbirth, especially placentas [1].

In prospective observational studies of pregnant woman, Q fever has been associated inconsistently with miscarriage [3, 4], preterm birth [5–7], or low birthweight [7], and infrequently with foetal death [8], or congenital malformations [8]. These adverse pregnancy outcomes (APOs) have been associated both with acute and persistent Q fever infections [9]. They are likely the consequence of detrimental placental immune cell responses overcoming the normal host proinflammatory trophoblast cell program, whilst the human trophoblast is believed to serve as a niche for bacterial replication [10]. Notwithstanding, the causal relationship between exposure to Q fever and APOs remains elusive given discrepancies between case series and observational studies.

Following the observation of Q fever endocarditis [11], peaks of prematurity and unexplained foetal deaths in birth registries, and in the preparation of a serosurvey among parturient women (*Jaubert* et al.*, under review*), we conducted a prospective follow-up study to assess the cumulative incidence of APOs of unknown origin associated with *C. burnetii* infection. Our secondary objective was to evaluate the contribution of acute Q fever infection to APOs.

## Methods

### Setting and population

La Réunion is a small tropical island (2512 km^2^), located in the South Western Indian ocean, 700 km east of Madagascar. Landscapes are very contrasted with a mountainous centre separating a humid “windward” east coast from a dry “leeward” west coast. The domestic animal populations are comprised of roughly 40,000 cattle, 30,000 goats and 2000 sheep, mainly based in the West and the South microregions [2]. Coastal areas are the most densely populated and host approximately 80% of the 816,000 residents.

Between May 1 and October 31, 2013, all pregnant women presenting at the regional perinatal health care centre of Saint Pierre for an unexplained early (< 12 weeks) or late (12 to 21 weeks) miscarriage, stillbirth (intrauterine foetal death ≥22 weeks) preterm birth (PTB, < 37 weeks) or small-for-gestational age child (SGA, birthweight <10th percentile), were proposed the addition of a Q fever workup in addition to the usual data collection of a birth registry [12, 13]. Women were enrolled either prospectively, when the APO event was suspected (e.g., preterm labour, poor growth of uterine height), or retrospectively, when the APO event had occurred.

### Laboratory methods

Sera were tested using an indirect fluorescent antibody (IFA) assay with commercially available antigens for *C. burnetii* (*C. burnetti* I + II IFA IgG/IgM/IgAt®, Vircell, Grenade, Spain).

Seropositivity was defined as a phase 2 or phase 1 IgG titre ≥1:64 with or without phase 2/1 IgM ≥ 1:48. Acute Q fever was defined as a high phase 2 IgG titre ≥1:256 (compatible with a recent or an active infection [3]), or detection of *C. burnetii* genome on miscarriage products and placentas. These conservative thresholds were chosen to fulfil the National Reference Centre requirements and minimize the false positives [14]. Persistent Q fever was defined as a phase 1 to phase 2 IgG ratio > 1 in the absence of IgM antibodies [15]. Women were proposed serology follow-up to check for seroconversion (4-fold increase in titres between 2 paired samples) as done in standard care.

Bacterial DNA within birth products was detected by real-time polymerase chain reaction (PCR) amplification of the IS1111 region of the C*. burnetti* genome. The DNA extraction, preparation of plasmid standards, PCR assays and probe analysis were performed using Klee’s protocol [16]. Primers and probes were designed using the Primer Express software® (Applied Biosystems, Darmstadt, Germany) and purchased from TIB Molbiol (Berlin, Germany).

Biological plausibility was defined as a positive RT-PCR or the seroconversion of phase 2 IgG. The relationship between exposure to Q fever and APOs was deemed causal when temporality and biological plausibility criteria were met and TORCH (*Toxoplasma gondii*, other infections, rubella, cytomegalovirus, and herpes simplex virus [HSV]-2 or neonatal herpes simplex) pathogens ruled out.

### Statistical analysis

Cumulative incidence rates of APOs were measured per 1000 pregnant women within the participant sample and extrapolated to the total number of APOs in the population observed during the study period using resampling weights based on demographics to minimize selection and misclassification biases (Additional file 1).

Miscarriage, stillbirth, preterm birth, small-for-gestational age as well as a composite outcome of all these APOs were compared according to *C. burnetii* exposure using chi2 or Fisher exact tests. In addition, incidence rate ratios (IRR) of each Q fever related APO were estimated using Poisson regression models for dichotomous outcomes with the robust variance option adjusted for hypertensive disorders, diabetes (gestational or pre-gestational), and maternal addictions (smoking or alcohol). Attributable risk percent (i.e., etiologic fractions) among the exposed and population attributable fractions were generated to estimate the contribution of *C. burnetii* exposure to APOs.

All these analyses were performed using Stata 14.2® (StataCorp, College Station, TX, USA). For all estimations, a *P* value < 0.05 was considered significant.

## Results

**Fig. 1:**
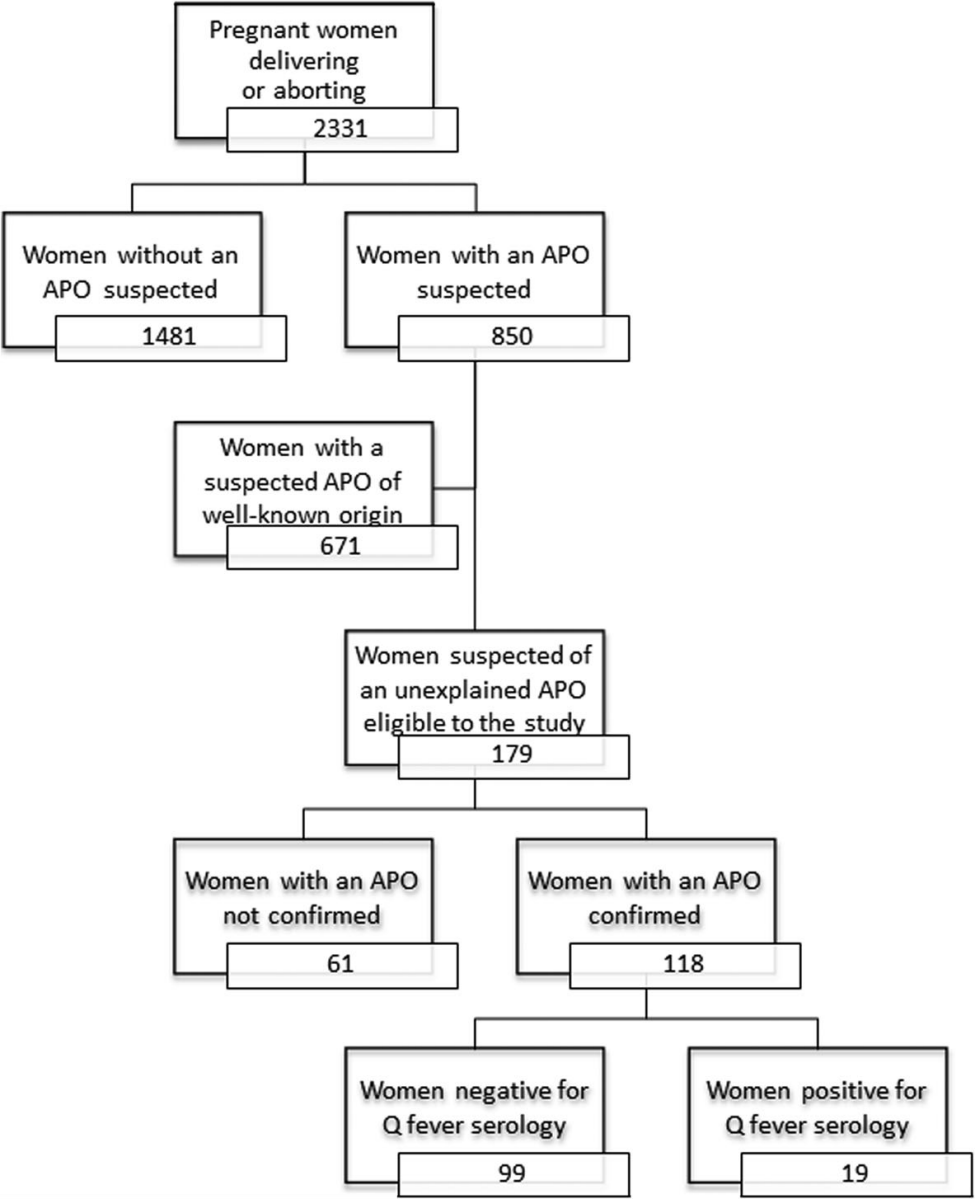
Distribution of the study population

Between May 1 and October 31, 2013, 2331 pregnant women gave birth or aborted within the level-3 maternity. Of these, 850 (36.4%) were suspected of an APO (Fig. 1). Among these, the suspected APO was linked to a known cause of perinatal complication for 668 women and was unexplained for 182 other women. Among unexplained suspected APOs, 179 pregnant women consented to be investigated for Q fever and were enrolled in the follow-up study, of whom 118 (65.8%) confirmed an APO. The participant women were representative of the reproductive population in terms of demographics, pregnancy-related hypertensive disorders, diabetes, or foetal gender but were less likely to smoke or drink alcohol, and more likely to carry a multiple pregnancy (Additional file 1: Table S1). Participant women were also indistinguishable from other women presenting APOs on the aforementioned factors (Additional file 1: Table S2).

Of the 179 participants, 19 were seropositive (phase 2 IgG titre ≥1:64 in all cases, phase 2 IgM ≥ 1:48 also in three cases) and 10 presented a profile indicative of an acute infection (phase 2 IgG ≥ 1:256, of whom the three with phase 2 IgM ≥ 1:48). It was worth noting that Q fever genome was not recovered from all the 10 birth products sampled from early or late miscarriages. Except patient n°1, who exhibited fever and mild hepatitis, all parturient women were asymptomatic, and the diagnosis was made incidentally at the occasion of the APO event. No persistent infection was identified upon follow-up. The serological profiles and perinatal complications of the seropositive women are presented in Table 1.

**Table 1:** Serology titres and perinatal complications of 19 women with Q fever among 179 pregnant women with adverse pregnancy outcomes (APO), Saint Pierre, May to October 2013

Patient	Age range (years)	Phase 2 IgM	Phase 2 IgG	Phase 1 IgG	APO (weeks)
1	35 to 40	48	256	0	LPTB (36 weeks)
2	31 to 35	96	256	0	EM (9 weeks)
3	15 to 20	0	64	0	SGA + OA (39 weeks)
4	31 to 35	0	128	0	EM (10 weeks)
5	21 to 25	192	1024	128	EM (5 weeks)
6	41 to 45	0	512	128	EM (8 weeks)
7	21 to 25	0	256	0	NGA (39 weeks)
8	26 to 30	0	1024	0	EM (8 weeks)
9	36 to 40	0	256	0	HM + EM (8 weeks)
10	16 to 20	0	256	0	IUFD+LPTB(36 weeks)
11	16 to 20	0	64	0	EM (6 weeks)
12	31 to 35	0	64	0	LM (21 weeks)
13	16 to 20	0	64	0	LPTB+SGA (36 weeks)
14	41 to 45	0	128	0	EM (9 weeks)
15	31 to 35	0	256	0	IUFD+VPTB (32 weeks)
16	36 to 40	0	128	0	EM (9 weeks)
17	26 to 30	0	64	0	VPTB+SGA (31 weeks)
18	36 to 40	0	256	0	SGA (39 weeks)
19	21 to 25	0	128	0	SGA (37 weeks)

*EM* early miscarriage (aka spontaneous abortion< 12 weeks of gestation), *HM* hydatiform mole, *LM* late miscarriage (aka, spontaneous abortion 12 to 21 weeks, or birthweight< 500 g.), *IUFD* intrauterine foetal death (aka stillbirth, ≥ 22 weeks or birthweight ≥500 g.), *VPTB* very preterm birth (22 to 32 weeks), *LPTB* late preterm birth (33 to 36 weeks), *NGA* normal for gestational age, *SGA* small for gestational age (intrauterine growth restriction; birthweight <10th percentile), *OA* oligohydramnios

Of the 21 APOs observed within the 19 seropositive women, early miscarriage (*n* = 9) was the most common perinatal complication followed by SGA (*n* = 5), PTB (*n* = 3), stillbirth (*n* = 2), late miscarriage and oligohydramnios (*n* = 1). For three women with an acute Q fever (n°1, n°2, n°5), the chronology between the onset of the infection and the APO (i.e., temporality) with respect to the kinetics of antibodies (i.e., seroconversion ensuring biological plausibility) suggested causality in the pathogenesis of the complication.

The incidence of Q fever related APO in the reproductive population was estimated at between 2.4‰ and 5.4‰ (1.3 to 4.3‰ in the study sample), whether causality was requested or not, which was in the observed range for TORCH pathogens. Miscarriage featured almost half of this burden (Additional file 1: Table S3).

*C. burnetii* exposure or acute Q fever was independently associated with a composite outcome of APOs in a model controlling for major confounders such as pregnancy-related hypertensive disorders, diabetes or maternal addictions (Table 2).

**Table 2:** Adverse pregnancy outcomes associated with Q fever seropositivity in bivariate and multivariate analysis, among 179 pregnant women, Saint Pierre, Reunion island, May to October 2013

Adverse pregnancy outcomes	n	%	*P value*	Crude IRR	95% CI	Adjusted IRR^b^	95% CI
A. Exposure variable: Coxiella burnetii Phase 2 IgG ≥ 1:64							
Composite outcome^a^			0.004				
In exposed	18 / 19	94.7		1.53	1.30–1.80	1.55	1.31–1.84
In unexposed	99 / 160	61.9		1		1	
Miscarriage			0.004				
In exposed	10 / 19	52.6		2.34	1.39–3.92	2.33	1.48–3.67
In unexposed	36 / 160	22.5		1		1	
Stillbirth			0.287				
In exposed	2 / 19	10.5		2.11	0.48–9.23	1.70	0.43–6.70
In unexposed	8 / 160	5.0		1		1	
Preterm birth			0.568				
In exposed	5 / 19	26.3		1.24	0.55–2.79	1.38	0.72–2.62
In unexposed	34 / 160	21.3		1		1	
Small-for-gestational age			0.959				
In exposed	5 / 19	26.3		0.98	0.44–2.17	1.03	0.49–2.13
In unexposed	43 / 160	26.9					
B. Exposure variable: Coxiella burnetii Phase 2 IgG ≥ 1:256 or Phase 2 IgM ≥ 1:48							
Composite outcome^a^			0.168				
In exposed	9 / 10	90.0		1.41	1.11–1.78	1.47	1.15–1.89
In unexposed	108/169	63.9		1		1	
Miscarriage			0.070				
In exposed	5 / 10	50.0		2.06	1.04–4.05	1.78	0.94–3.39
In unexposed	41 / 169	24.3		1		1	
Stillbirth			0.099				
In exposed	2 / 10	20.0		4.23	1.02–17.41	3.19	0.92–11.00
In unexposed	8 / 169	4.7		1		1	
Preterm birth			0.456				
In exposed	3 / 10	30.0		1.41	0.52–3.80	1.75	0.71–4.31
In unexposed	36 / 169	21.3		1		1	
Small-for-gestational age			0.292				
In exposed	1 / 10	10.0		0.35	0.05–2.36	0.42	0.06–2.87
In unexposed	47/169	27.8		1			

Data are numbers, seropositive rates (%), crude and adjusted incidence rate ratios (IRR) and 95% confidence intervals (95% CI). *P* values are given for Pearson chi2 tests. ^a^Miscarriage, stillbirth, or preterm birth, or small-for-gestational age. ^b^Multivariate Poisson regression model with robust variance option adjusted on hypertensive pregnancy disorders, diabetes (gestational or pregestational), and maternal addictions (smoking or alcohol)

Pregnant women with *C. burnetii* antibodies were more likely to suffer a miscarriage, and there was trend to increase risk for stillbirth at the threshold defining acute Q fever.

Importantly, both risks for miscarriage and stillbirth were highly attributable to the exposure (Table 3), which supports the involvement of *C. burnetii* in their pathogenesis. The population attributable fraction for Q fever exposure was 12%, which means that if Q fever had been fully treatable with antibiotics, the burden of miscarriage should have been less than 88% of that observed.

**Table 3:** Contribution of Q fever infection to adverse pregnancy outcomes among 179 pregnant women with perinatal complications, Saint Pierre, Reunion island, May to October 2013

Adverse pregnancy outcomes	ARP (%)	95% CI	PAF (%)
A. Exposure variable: Coxiella burnetii Phase 2 IgG ≥ 1:64			
Composite outcome^a^	34.7	23.2–44.4	5.3
Miscarriage	57.3	28.5–74.4	12.4
Stillbirth	52.5	−1.07 - 89.1	10.5
B. Exposure variable: Coxiella burnetii Phase 2 IgG ≥ 1:256 or Phase 2 IgM ≥ 1:48			
Composite outcome^a^	29.0	10.1–43.9	2.2
Miscarriage	51.5	4.7–75.3	5.6
Stillbirth	76.3	0.2–94.2	15.3

Data are attributable fractions among the exposed (ARP or etiologic fractions), 95% confidence intervals (95% CI), and population attributable fractions (PAF). ^a^Miscarriage, stillbirth, or preterm birth, or small-for-gestational age

## Discussion

This prospective study suggests a potential burden of Q fever to APOs in a setting of putative endemic transmission [2], although the documentation of human cases and the understanding of transmission pathways in the community remain uncomplete. Despite being conducted over a very short period of observation and the a selection of women at risk minimizing the actual proportion of infected women, recent or acute *C. burnetii* infections were deemed responsible of two miscarriages and one late preterm birth, which shed light on a possible reproductive health concern. In support of this finding, the cumulative incidences of Q fever associated APOs was estimated to reach a level that should be considered in daily obstetrical practice. Indeed, this burden was consistent with the figures reported either from endemic [7] or epidemic settings (Additional file 1: Table S3) [4, 5], and it was also coherent with what has been observed in Réunion island for TORCH pathogens [17].

This prospective study also supports the strong association between acute or recent Q fever and APOs, especially miscarriage, as previously found in a Spanish case control study [3]. Together with high attributable risk percent suggesting the contribution of Q fever in pathogenesis, our findings argue a causative role for C. *burnetii* infection in miscarriage, and to a lesser extent, intrauterine foetal death, as proposed in a meta-analysis [8]. In this latter study, spontaneous abortions were pooled together with stillbirths and early postnatal deaths, which makes the contribution of Q fever to each independent outcome unclear. Our findings are however in agreement with a Danish cohort study and studies from the Netherlands, which suggest weakening the putative association between Q fever exposure and preterm birth [5, 6, 18] or small-for-gestational age children [5, 6].

Importantly, we have shown that in the endemic context of Reunion island, acute Q fever may lead to APOs. Although it has not been recommended in post-epidemic situations [19], in our endemic context we advocate that pregnant women with a previous episode of miscarriage or stillborn child or presenting an environmental or occupational risk of Q fever, be screened before conception, or early in pregnancy, and treated with cotrimoxazole (and acid folic supplementation) for at least 5 weeks if a seroconversion with phase 2 IgG antibodies occurs, or until delivery if phase 1 IgG antibodies are present, in order to avoid potential harms to the foetus [20] and/or progression to persistent infection [21]. Furthermore, all pregnant women could be informed of the potential risks of Q fever, as already occurs for arboviral infections such as Zika virus to improve the awareness and allow then to limit their exposition if possible, for instance by keeping away from farms and avoiding direct contact with ruminants. In case of fever of unknown origin, pneumonia, hepatitis, endocarditis, unexplained perinatal complication, or overt exposure during pregnancy, we endorse investigating acute Q fever as a possible diagnosis with close monitoring of *Coxiella burnetii* antibodies, and to treat when appropriate [21]. Because placental inoculation may ensue, although PCRs were negative in our study, we encourage to complete the workup dedicated to foetal issues with PCRs targeting amniotic fluids, placentas and birth products. Because person-to-person transmission of Q fever has been suspected in a maternity ward [22], we strongly recommend precautions in birth product manipulations and quarantine of the infected pregnant woman. Because persistent infection (e.g., endocarditis, vasculitis, osteoarthritis, lymphadenitis) may complicate Q fever onset through pregnancy, ante or postpartum, we propose the long-term follow-up of parturient women and screening of secondary locations [21].

## Limitations

### Limitations of the study

First, the presented work was an exploratory investigation performed in the preparation of an academically funded serosurvey leading insufficient power to detect an effect of Q fever on stillbirth while adjusting for relevant confounders. Second, the selection of a control population at risk has certainly diminished the magnitude of the effect of Q fever on APOs. This was a practical conservative option given the impossibility to conduct both simultaneously the serosurvey (under the rules of biomedical research ethics) and this follow-up study (under the rules of standard care research). We believe however this stringent methodological option (known as tip of iceberg epidemiology) makes the results from our local study that more interesting, the real burden being likely underestimated.

In support to the eventuality of underreporting, there is a potential for Q fever to be associated with other pregnancy outcomes, such as oligohydramnios, hydramnios, or preterm premature rupture of membranes, as suggested by several case series [9, 23].

## Conclusion

Q fever is circulating on Reunion island, including among pregnant women. Given that specific strains have been found to be associated with different clinical manifestations and given the increased risk of miscarriage in our cohort study, it would now be interesting to identify whether the circulating strain is abortive, as that harbouring the QpDV plasmid [20].

As for mitigation measures aimed at limiting the burden of Q fever on reproduction, pregnant women should be kept away from farms to rule out airborne transmission, avoid direct contact with ruminants, or to consume fresh farm products. In addition, whenever possible, at risk women of childbearing age should be screened before conception or early in pregnancy and be treated with antibiotics to prevent potential harm to the foetus. Our findings are of particular interest to public health stakeholders and policy makers based in countries where Q fever is endemic to protect communities, especially in rearing areas.

## Supplementary information

**Additional file 1.** Methodological appendix; **Table S1** Maternal and foetal characteristics in the South Réunion island reproductive population and the study population; **Table S2** Adverse pregnancy outcomes (APO) in the eligible population and the study population according to maternal and foetal characteristics; Supplemental findings; **Table S3** Adverse pregnancy outcomes associated with Q fever seropositivity in bivariate and multivariate analysis; **Table S4** Cumulative seroincidence rates of adverse pregnancy outcomes (APOs) associated with positive Q fever serology (any cut-off) in prospective observational studies reported in the literature.

## Data Availability

The dataset generated and/or analysed during the current study are not publicly available due to anonymity policy issues but are available from the corresponding author on reasonable request.

## Abbreviations

95%CI: 95% confidence interval
APOs: Adverse pregnancy outcomes
ARP: Attributable risk percent
IFA: Indirect fluorescent antibody (alternatively taken as immunofluorescent assay)
IgA: Immunoglobulin A
IgG: Immunoglobulin G
IgG2: Phase 2 immunoglobulin G
IgM: Immunoglobulin M
IRR: Incidence proportion ratio
PAF: Population attributable fraction
TORCH: *Toxoplasma gondii*, Other (syphilis, varicella-zoster, parvovirus B19), Rubella, Cytomegalovirus, Herpes)
