# Supplementary material for: Pregnancy outcomes of Q fever: prospective follow-up study on Reunion island
Source: BMC Infect Dis. 2019 Nov 27;19:1001. doi: 10.1186/s12879-019-4619-6 (PMC6880502; doi:10.1186/s12879-019-4619-6)
Supplement: Supplementary file 1 — Additional file 1. Methodological appendix; Table S1 Maternal and foetal characteristics in the South Réunion island reproductive population and the study population; Table S2 Adverse pregnancy outcomes (APO) in the eligible population and the study population according to maternal and foetal characteristics; Supplemental findings; Table S3 Adverse pregnancy outcomes associated with Q fever seropositivity in bivariate and multivariate analysis; Table S4 Cumulative seroincidence rates of adverse pregnancy outcomes (APOs) associated with positive Q fever serology (any cut-off) in prospective observational studies reported in the literature. [file 12879_2019_4619_MOESM1_ESM.docx]

**Additional file 1:** Methodological appendix; **Table S1** Maternal and foetal characteristics in the South Réunion island reproductive population and the study population; **Table S2** Adverse pregnancy outcomes (APO) in the eligible population and the study population according to maternal and foetal characteristics; Supplemental findings; **Table S3** Adverse pregnancy outcomes associated with Q fever seropositivity in bivariate and multivariate analysis; **Table S4.** Cumulative seroincidence rates of adverse pregnancy outcomes (APOs) associated with positive Q fever serology (any cut-off) in prospective observational studies reported in the literature.

**Methodological appendix.**

The number of adverse pregnancy outcome events associated with acute infections (Ysi) observed in the study sample (s) was extrapolated to the reproductive population (p) using resampling weights based on maternal demographics (age, place of birth, marital status, education, occupation and parity) to minimize selection and misclassification biases, as follows:

Yp_i_ _=_ _Ysi_ + (Yp_0_ - Ys_0_)× ^ρ^

Ys_0_

where Ypi is the expected number of outcome events in the infected (unknown) within the reproductive population, Yp0, the number of outcomes events in the reproductive population subtracted of Ysi (observed), Ys0, the number of outcomes events in the uninfected study population (observed) and ρ, a multiplicative factor corresponding to the proportion of outcome events in the infected population after reweighting on demographics.

| **Table S1. Maternal and foetal characteristics in the South Réunion island reproductive population and the study population, Saint Pierre, Reunion island, May to October 2013** | | | | | | |
| --- | --- | --- | --- | --- | --- | --- |
|  | **Reproductive population** | | **Study population** | | **QF -** | **QF+** |
| **Variables** | **N** | **%** | **n** | **%** | **n** | **n** |
| **Maternity centre** |  |  |  |  |  |  |
| Level-3, Saint Pierre | 2,152 | 100 | 179 | 100 | 159 | 19 |
| **Age** |  |  |  |  |  |  |
| <25 years | 748 | 35.2 | 58 | 32.4 | 53 | 5 |
| 25-30 years | 676 | 31.8 | 51 | 28.5 | 47 | 4 |
| 31-47 years | 699 | 32.9 | 70 | 39.1 | 60 | 10 |
| **Place of birth** |  |  |  |  |  |  |
| Reunion | 1,621 | 78.8 | 84 | 86.6 | 75 | 9 |
| Indian ocean | 202 | 9.8 | 5 | 5.2 | 5 | 0 |
| Metropolitan France | 235 | 11.4 | 8 | 8.2 | 8 | 0 |
| **Marital Status** |  |  |  |  |  |  |
| In couple | 1,289 | 62.7 | 45 | 46.9 | 40 | 5 |
| Celibacy | 767 | 37.3 | 51 | 53.1 | 47 | 4 |
| **Education** |  |  |  |  |  |  |
| Primary school | 70 | 3.4 | 2 | 2.1 | 2 | 0 |
| Middle school | 624 | 30.7 | 38 | 39.2 | 36 | 2 |
| High school | 761 | 37.5 | 35 | 36.1 | 30 | 5 |
| University | 577 | 28.4 | 22 | 22.7 | 19 | 3 |
| **Occupation** |  |  |  |  |  |  |
| Unemployed | 1,456 | 70.7 | 65 | 67.0 | 59 | 6 |
| Farmer | 7 | 0.3 | 0 | 0.0 | 0 | 0 |
| Other work | 597 | 29.0 | 32 | 33.0 | 28 | 4 |
| **Parity** |  |  |  |  |  |  |
| Nullipara | 770 | 37.2 | 45 | 46.4 | 38 | 7 |
| Primipara | 638 | 30.9 | 23 | 23.7 | 21 | 2 |
| Multipara | 660 | 31.9 | 29 | 29.9 | 28 | 1 |
| **Addiction (smoking or alcohol)** | *P* value = 0.023 | | | |  |  |
| Yes | 283 | 13.1 | 13 | 7.3 | 12 | 1 |
| No | 1,869 | 86.9 | 166 | 92.7 | 148 | 18 |
| **Pregnancy hypertensive disorders** | |  |  |  |  |  |
| Yes | 112 | 5.2 | 13 | 7.3 | 11 | 2 |
| No | 2,040 | 94.8 | 166 | 92.7 | 149 | 17 |
| **Diabetes (gestational or pregestational)** | |  |  |  |  |  |
| Yes | 263 | 12.2 | 18 | 10.1 | 17 | 1 |
| No | 1,889 | 87.8 | 161 | 89.9 | 143 | 18 |
| **Multiple pregnancy** | *P* value = 0.031 | | | |  |  |
| Yes | 35 | 1.7 | 5 | 5.2 | 3 | 2 |
| No | 2,036 | 98.3 | 92 | 94.8 | 85 | 7 |
| **Foetal gender** |  |  |  |  |  |  |
| Male | 1,034 | 49.8 | 49 | 51.0 | 45 | 4 |
| Female | 1,041 | 50.2 | 47 | 49.0 | 42 | 5 |
| Data are numbers (N and n) or percentages for the reproductive population, the study population, and exposure groups. *P* values are given for chi2 or Fisher exact tests comparing col.1 *vs* col.3, and col. 5 *vs* col.6. | | | | | | |
| **Table S2. Adverse pregnancy outcomes (APO) in the eligible population and the study population according to maternal and foetal characteristics, Saint Pierre, Reunion island, May to October 2013** | | | | | | |
|  | **Eligible population**  **with APO** | | **Study population**  **with APO** | | **QF -** | **QF+** |
| **Variables** | **N** | **%** | **n** | **%** | **n** | **n** |
| **Maternity centre** |  |  |  |  |  |  |
| Level-3, Saint Pierre | 732 | 100 | 118 | 100 | 99 | 19 |
| **Age** |  |  |  |  |  |  |
| <25 years | 251 | 35.2 | 37 | 31.4 | 32 | 5 |
| 25-30 years | 216 | 30.2 | 27 | 22.9 | 23 | 4 |
| 31-47 years | 247 | 34.6 | 54 | 45.7 | 44 | 10 |
| **Place of birth** |  |  |  |  |  |  |
| Reunion | 524 | 80.4 | 61 | 85.9 | 52 | 9 |
| Indian ocean | 58 | 8.9 | 4 | 5.6 | 4 | 0 |
| Metropolitan France | 70 | 10.7 | 6 | 8.5 | 6 | 0 |
| **Marital Status** |  |  |  |  |  |  |
| In couple | 404 | 61.9 | 36 | 51.4 | 31 | 5 |
| Celibacy | 249 | 38.1 | 34 | 48.6 | 30 | 4 |
| **Education** |  |  |  |  |  |  |
| Primary school | 19 | 3.0 | 1 | 1.4 | 1 | 0 |
| Middle school | 195 | 30.4 | 30 | 42.3 | 28 | 2 |
| High school | 230 | 35.9 | 27 | 38.0 | 22 | 5 |
| University | 197 | 30.7 | 13 | 18.3 | 10 | 3 |
| **Occupation** |  |  |  |  |  |  |
| Unemployed | 460 | 70.7 | 50 | 70.4 | 44 | 6 |
| Farmer | 2 | 0.3 | 0 | 0.0 | 0 | 0 |
| Other work | 189 | 29.0 | 21 | 29.6 | 17 | 4 |
| **Parity** |  |  |  |  |  |  |
| Nullipara | 283 | 43.3 | 34 | 47.9 | 27 | 7 |
| Primipara | 186 | 28.5 | 16 | 22.5 | 14 | 2 |
| Multipara | 184 | 28.2 | 21 | 29.6 | 20 | 1 |
| **Addiction (smoking or alcohol)** |  |  |  |  |  |  |
| Yes | 116 | 15.9 | 11 | 9.3 | 10 | 1 |
| No | 616 | 84.1 | 107 | 90.7 | 89 | 18 |
| **Pregnancy hypertensive disorders** | |  |  |  |  |  |
| Yes | 65 | 8.9 | 12 | 10.2 | 10 | 2 |
| No | 667 | 91.1 | 106 | 89.8 | 89 | 17 |
| **Diabetes (gestational or pregestational)** | |  |  |  |  |  |
| Yes | 66 | 9.0 | 15 | 12.7 | 14 | 1 |
| No | 666 | 91.0 | 103 | 87.3 | 85 | 18 |
| **Multiple pregnancy** |  |  |  |  |  |  |
| Yes | 28 | 4.3 | 5 | 7.0 | 3 | 2 |
| No | 627 | 95.7 | 66 | 93.0 | 58 | 7 |
| **Foetal gender** |  |  |  |  |  |  |
| Male | 278 | 42.4 | 37 | 52.1 | 33 | 4 |
| Female | 378 | 57.6 | 34 | 47.9 | 29 | 5 |
| Data are numbers (N and n) or percentages for the reproductive population, the study population, and exposure groups. *P* values are given for chi2 or Fisher exact tests comparing col.1 *vs* col.3, and col. 5 *vs* col.6. | | | | | | |

**Supplemental findings**

Expected number of adverse pregnancy outcomes associated with infection in the reproductive population

**Case situation 1.**

Y_si_ = 3 (number observed of probable or certain acute infections)

Y_p0_ = 850 – 19 = 831

Ys_0_ = 118 – 19 = 99

ρ = 0.1836 ÷ 0.6197 = 0.29627239

Total case number : Y_pi_ = Y_si_ + [(831 - 99) ÷ 99 × 0.29627239] = 3 + 2.2 = 5.2

Cumulative incidence rate (CIR): 5.2 × 1,000 ÷ 2,331 = 2.2 outcome events per 1,000 women.

**Case situation 2.**

Ysi = 10 (number observed of possible acute infections)

Yp0 = 850 – 19 = 831

Ys0 = 118 – 19 = 99

ρ = 0.1836 ÷ 0.6197 = 0.29627239

Total case number : Y_pi_ = Y_si_ + [(831 - 99) ÷ 99 × 0.29627239] = 10 + 2.2 = 12.2

Cumulative incidence (CIR): 12.2 × 1,000 ÷ 2331 = 5.2 outcome events per 1,000 women.

| **Table S3 Adverse pregnancy outcomes associated with Q fever seropositivity in bivariate and multivariate analysis, among 179 pregnant women, Saint Pierre, Reunion island, May to October 2013** | | | | | | | | | | | | |  |
| --- | --- | --- | --- | --- | --- | --- | --- | --- | --- | --- | --- | --- | --- |
| **Adverse** | **n** | | **%** | | ***P value*** | **Crude IRR** | | **95% CI** | | **Adjusted IRR^#^** | | **95% CI** | |
| **pregnancy outcomes** | 1. ***Exposure variable : Coxiella burnetii Phase 2 IgG ≥ 1:64*** | | | | | | | | | | | |  |
| **Composite outcome*** |  |  | | 0.004 | |  |  | |  | |  | |  |
| In exposed | 18 / 19 | 94.7 | |  | | 1.53 | 1.30 - 1.80 | | 1.55 | | 1.31 - 1.84 | |  |
| In unexposed | 99 / 160 | 61.9 | |  | | 1 |  | | 1 | |  | |  |
| **Miscarriage** |  |  | | 0.004 | |  |  | |  | |  | |  |
| In exposed | 10 / 19 | 52.6 | |  | | 2.34 | 1.39 - 3.92 | | 2.33 | | 1.48 - 3.67 | |  |
| In unexposed | 36 / 160 | 22.5 | |  | | 1 |  | | 1 | |  | |  |
| **Stillbirth** |  |  | | 0.287 | |  |  | |  | |  | |  |
| In exposed | 2 / 19 | 10.5 | |  | | 2.11 | 0.48 - 9.23 | | 1.70 | | 0.43 - 6.70 | |  |
| In unexposed | 8 / 160 | 5.0 | |  | | 1 |  | | 1 | |  | |  |
| **Preterm birth** |  |  | | 0.568 | |  |  | |  | |  | |  |
| In exposed | 5 / 19 | 26.3 | |  | | 1.24 | 0.55 - 2.79 | | 1.38 | | 0.72 - 2.62 | |  |
| In unexposed | 34 / 160 | 21.3 | |  | | 1 |  | | 1 | |  | |  |
| **Small-for-gestational age** | |  | | 0.959 | |  |  | |  | |  | |  |
| In exposed | 5 / 19 | 26.3 | |  | | 0.98 | 0.44 - 2.17 | | 1.03 | | 0.49 - 2.13 | |  |
| In unexposed | 43 / 160 | 26.9 | |  | |  |  | |  | |  | |  |
|  | ***B. Exposure variable : Coxiella burnetii Phase 2 IgG ≥ 1:256 or Phase 2 IgM ≥ 1:48*** | | | | | | | | | | | |  |
| **Composite outcome*** |  |  | | 0.168 | |  |  | |  | |  | |  |
| In exposed | 9 / 10 | 90.0 | |  | | 1.41 | 1.11 - 1.78 | | 1.47 | | 1.15 - 1.89 | |  |
| In unexposed | 108/169 | 63.9 | |  | | 1 |  | | 1 | |  | |  |
| **Miscarriage** |  |  | | 0.070 | |  |  | |  | |  | |  |
| In exposed | 5 / 10 | 50.0 | |  | | 2.06 | 1.04 - 4.05 | | 1.78 | | 0.94 - 3.39 | |  |
| In unexposed | 41 / 169 | 24.3 | |  | | 1 |  | | 1 | |  | |  |
| **Stillbirth** |  |  | | 0.099 | |  |  | |  | |  | |  |
| In exposed | 2 / 10 | 20.0 | |  | | 4.23 | 1.02-17.41 | | 3.19 | | 0.92-11.00 | |  |
| In unexposed | 8 / 169 | 4.7 | |  | | 1 |  | | 1 | |  | |  |
| **Preterm birth** |  |  | | 0.456 | |  |  | |  | |  | |  |
| In exposed | 3 / 10 | 30.0 | |  | | 1.41 | 0.52 - 3.80 | | 1.75 | | 0.71 - 4.31 | |  |
| In unexposed | 36 / 169 | 21.3 | |  | | 1 |  | | 1 | |  | |  |
| **Small-for-gestational age** | |  | | 0.292 | |  |  | |  | |  | |  |
| In exposed | 1 / 10 | 10.0 | |  | | 0.35 | 0.05 - 2.36 | | 0.42 | | 0.06 - 2.87 | |  |
| In unexposed | 47/169 | 27.8 | |  | | 1 |  | |  | |  | |  |
| Data are numbers, seropositive rates (%), crude and adjusted incidence rate ratios (IRR) and 95% confidence intervals (95% CI). *P* values are given for Pearson chi2 tests. *Miscarriage, stillbirth, or preterm birth, or small-for-gestational age. ^#^Multivariate Poisson regression model with robust variance option adjusted on hypertensive pregnancy disorders, diabetes (gestational or pregestational), and maternal addictions (smoking or alcohol) | | | | | | | | | | | | |  |

| **Table S4. Cumulative seroincidence rates of adverse pregnancy outcomes (APOs) associated with positive Q fever serology (any cut-off) in prospective observational studies reported in the literature** | | | | | |
| --- | --- | --- | --- | --- | --- |
| **Studies,**  **Years ^reference^** | **La Réunion (2013)** | **La Réunion (2013)** | **Canada**  **(1997-1998)^7^** | **Danemark**  **(1996-2002)^4,6^** | **Netherlands**  **(2007)^5^** |
| Epidemiological context | Endemic (observed) | Endemic (estimated) | Endemic  (observed) | Endemic  (observed) | Post-epidemic  (observed) |
| Any APOs | 8.6‰ | 9.5‰ | ? | 18.7‰ | 5.5‰ |
| Miscarriage | 3.9‰ | 4.8‰ | - | 2.3‰ | - |
| Stillbirth | 0.9‰ | 1.8‰ | 1.7‰ | - | 0‰ |
| Preterm birth | 1.3‰ | 2.2‰ | 5.2‰ | 3.5‰ | 1.8‰ |
| Small-for-gestational age | 2.1‰ | 3.1‰ | ? | 10.5‰ | 3.0‰ |
